# Trends and socioeconomic inequalities in self-rated health in Japan, 1986–2016

**DOI:** 10.1186/s12889-021-11708-6

**Published:** 2021-10-08

**Authors:** Hirokazu Tanaka, Johan P. Mackenbach, Yasuki Kobayashi

**Affiliations:** 1 Department of Public Health, Erasmus University Medical Center, 3000 CA Rotterdam, The Netherlands; 2 Department of Public Health and Occupational Medicine, Graduate School of Medicine, Mie University, Tsu, Mie 514-8507 Japan; 3 Department of Public Health, Graduate School of Medicine, The University of Tokyo, Bunkyo-ku, Tokyo, 113-0033 Japan

**Keywords:** Self-assessed health, Perceived health, Health surveys, Socioeconomic factors, Japanese population

## Abstract

**Introduction:**

Despite having very high life expectancy, Japan has relatively poor self-rated health, compared to other high-income countries. We studied trends and socioeconomic inequalities in self-rated health in Japan using nationally representative data.

**Methods:**

The Comprehensive Survey of Living Conditions was analyzed, every 3 years (*n* ≈ 0.6–0.8 million/year) from 1986 to 2016. Whereas previous studies dichotomized self-rated health as an outcome, we used four categories: very good, good, fair, and bad/very bad. Proportional odds ordinal logistic regression models are used, with ordinal scale self-rated health as an outcome, and age category, survey year and occupational class or educational level as independent variables.

**Results:**

In 2016, the age-adjusted percentages for self-rated health categorized as very good, good, fair, and bad/very bad, were 24.0, 17.1, 48.7, and 10.2% among working-age men, and 21.6, 17.5, 49.4, and 11.5% among working-age women, respectively. With 1986 as the reference year, the odds ratios (ORs) of less good self-rated health were lowest in 1995 (0.69; 95% Confidence Interval [95% CI]: 0.66–0.71 of working-age men), and highest in 2010 (1.23 [95% CI: 1.19–1.27]). The ORs of male, lower non-manual workers (compared to upper non-manual) increased from 1.12 (95% CI: 1.07–1.17) in 2010 to 1.20 (95% CI: 1.15–1.26) in 2016. Between 2010 and 2016, the ORs of working-age men with middle and low levels of education (compared to a high level of education) increased from 1.22 (95% CI: 1.18–1.27) to 1.34 (95% CI: 1.29–1.38), and from 1.47 (95% CI: 1.39–1.56) to 1.75 (95% CI: 1.63–1.88), respectively. The ORs of working-age women with middle and low levels of education also increased from 1.22 (95% CI: 1.17–1.28) to 1.32 (95% CI: 1.26–1.37), and from 1.74 (95% CI: 1.61–1.88) to 2.03 (95% CI: 1.87–2.21) during the same period.

**Conclusion:**

Japan has the unique feature that approximately 50% of the survey respondents rated their self-rated health as fair, but with important variations over time and between socioeconomic groups. In-depth studies of the role of socioeconomic conditions may shed light on the reasons for the high prevalence of poor self-rated health in Japan.

**Supplementary Information:**

The online version contains supplementary material available at 10.1186/s12889-021-11708-6.

## Introduction

Japan has achieved prolonged life expectancy and has been improving morbidity and disability for decades [1, 2]. Reducing socioeconomic inequalities in health is a key global public health priority; however, health inequalities are reportedly smaller in Japan than in Western countries [3]. International comparative studies have suggested that relationship between socioeconomic status and health-related behavior is weaker in Japan than in compared to European countries [4, 5]. Additionally, mortality inequalities among occupational classes—differences between upper non-manual workers and manual workers— were smaller in Japan than in European countries during the 2010s [6]. These data suggest that people in Japan enjoy a relatively high life expectancy with small variations in health between socioeconomic groups.

However, people of all social circumstances have not benefited uniformly in Japan in the last three decades because population health, particularly that of working-age men and women, was influenced by the restructuring of the national economy from the 1990s through the 2010s [7–11]. Japan’s economy underwent a long recession, the so-called “lost two decades” after the collapse of the “bubble economy” in the early 1990s. This resulted in instability in the Japanese labor market; the unemployment rate and the proportion of non-regular workers increased gradually during that period [12]. These circumstances may have contributed to long-term changes in health and health inequalities for the Japanese population. According to the statistics reported by the Organization for Economic Cooperation and Development (OECD), the Japanese population had relatively poor self-rated health in the 2010s compared to other high-income countries [13]. Although, as of 2017, 8.7% of adults across 35 OECD countries considered themselves to be in poor health, on average, the percentage in Japan was higher (14.1%), with relatively small income-based inequalities [13]. There are also some health indicators that show Japan has had a relatively poor record for health and health inequalities (e.g., high suicide rates and large socioeconomic inequalities in smoking prevalence) in recent decades [14, 15]. In this context, the overall picture of health inequalities in Japan is not yet clear.

In an effort to address the gap in the literature on population health and trends in health inequalities, we focused on the self-rated health of the Japanese population over three decades. Self-rated measures of health, shown to be reliable indicators of general health and mortality, are widely used in population health monitoring [16, 17]. Studies on Japan’s socioeconomic inequalities in self-rated health have been conducted using the nationwide Comprehensive Survey of Living Conditions (CSLC) with dichotomized self-rated health as an outcome [7–11]. One study reported that occupational inequalities in self-rated health increased somewhat after the 1997 Asian financial crisis [7]. Thereafter, trend analyses showed that socioeconomic inequalities in self-rated health by income level and occupational class had narrowed by 2007 [8, 9]. Furthermore, between 1986 and 2013, income-based inequalities in self-rated health have been negatively associated with unemployment during periods of high unemployment [10]. The associations between self-rated health and economic stagnation are still unclear because previous studies evidenced inconsistent associations not only in Japan [7–11] but worldwide [18–22].

The aim of this study was to investigate the details of changes in health and socioeconomic inequalities in health between 1986 and 2016.

## Methods

### Data

The CSLC, conducted by the Ministry of Health, Labor, and Welfare (MHLW) every 3 years from 1986 to 2016 (*n* ≈ 0.6–0.8 million/year), was used as the dataset for this study [23, 24]. In 2016, the CSLC interviewed all household members in 5530 randomly selected districts, from a total of 1,040,000 districts (approximately 50 households in each district), covering all prefectures in Japan. Finally, a total of 289,470 households were surveyed via distributed questionnaires (response rate: 77.6%) [23, 24]. Microdata were extracted and used with permission from the MHLW.

The household questionnaire included the demographic information for each household member (i.e., gender, age, marital status, health insurance, pension, dependents, working status, occupation, and educational background). The health questionnaire included questions about health status and medical treatment-seeking behavior (i.e., subjective symptoms, regular visits to hospital/clinics, difficulty in daily life, self-rated health status, mental health, physical examinations, alcohol consumption, and smoking status) [23].

### Definition of self-rated health

The CSLC assessed respondents’ self-rated health status with the single question: “How is your health, in general? Is it very good, good, fair, bad, or very bad?” Because “very bad” was highly unlikely to be selected [25], this study divided the five categories of self-rated health from the question into four categories of self-rated health: very good, good, fair, and bad/very bad. An ordinal scale of self-rated health, ranging from 0 = very good, 1 = good, 2 = fair, to 3 = bad/very bad, was defined as the outcome variable.

### Socioeconomic status

Socioeconomic status was assessed as both occupational class and educational level. Occupational class was divided into five categories: upper non-manual workers (i.e. professionals and managers), lower non-manual workers (i.e. clerical, service and sales workers), manual workers (i.e. crafts and related trade workers, semi-skilled and unskilled manual workers), farmers, and the self-employed. This classification followed the Erikson-Goldthorpe-Portocarero (EGP) scheme [26]. Educational level was divided into three categories: low (International Standard Classification of Education [ISCED]: 1, 2), middle (ISCED: 3, 4), and high (ISCED: 5–8). In Japan, “low” corresponds to elementary school/junior high school graduates, “middle” corresponds to high school graduates/technical or professional school graduates, and “high” corresponds to two-year college graduates and over [27]. The educational level was available from 2010. The details of the classifications and the distributions are shown in Additional file 1: Appendix Tables 1, 2, and 3.

### Statistical analysis

Gender and age were included in all analyses, with age divided into two age groups (25–64 years, 65–94 years), and weighted using the weight score provided by the MHLW. Occupational class was included in all analyses, for working-age men and women (25–64 years), during the study period. Educational levels were included in the analyses for both age groups, after the 2010 survey. Occupational class and educational level were not included in the same model, to avoid multicollinearity.

First, we used proportional odds ordinal logistic regression models, with ordinal scale self-rated health as an outcome, and age category (5 year age intervals) and occupational class (or educational level), to determine each variables’ probability of less good self-rated health [28, 29]. After fitting the model, the age-adjusted percentages of self-rated health were calculated by estimating the predicted probability of self-rated health, for each study participant, fixing occupational class (or educational level) at each categorical level and averaging over the sample; this is known as the marginal standardization form of predictive margins [30]. All the analyses were conducted for each survey year (Model 1).

Second, we included a dummy variable for the survey year into Model 1, as a covariate. The odds ratio (OR) for the four-level (0 = very good, 1 = good, 2 = fair, and 3 = bad/very bad) ordinal regression model shows a weighted average of three each OR when we increase the binary cut off point from one level to the next level; a larger OR indicates a higher probability of less good self-rated health, compared with the reference group or year [31]. After fitting the model, we applied marginal standardization to calculate the age-adjusted percentages of self-rated health, for the whole population, by survey year (Model 2: Pooled analysis).

Finally, we conducted a segmented regression analysis, based on Model 2, including the interaction terms between occupational class (or educational level) and survey year. The survey years were divided into three segments (1986–1995, 1995–2010, 2010–2016), because the trend of self-rated health changed in the years 1995 and 2010, according to the results of Model 2. The interaction terms were used to determine whether inequalities increased or decreased in each period (Model 3). We used Stata version 15/SE (StataCorp, College Station, TX, USA) for the analyses, and *p* values less than 0.05 were considered statistically significant.

## Results

### Sample population

Table 1 shows the analyzed survey participants’ demographic characteristics, covering all survey years. The sample populations in 1986, 1995, 2010, and 2016 were 203,913, 183,361, 130,938, and 128,787 for men and 219,590, 194,538, 140,212, and 136,789 for women, respectively. In 2016, the weighted proportions of upper non-manual workers, lower non-manual workers, manual workers, farmers, and self-employed were 38.0, 26.7, 23.5, 2.5, and 9.3% among working-age men (aged 25–64 years), respectively. The weighted proportions of upper non-manual workers, lower non-manual workers, manual workers, farmers, and self-employed were 25.9, 58.4, 10.4, 1.8, and 3.6% among working-age women, respectively. In 2016, the weighted proportions for individuals with high, middle, and low education levels were 43.2, 50.9, and 5.9% for men, and 40.1, 55.6, and 4.3% for women, respectively. The trends indicated more people with high socioeconomic status (both occupational class and educational class) were included in our study participants in the recent survey.

**Table 1:** Number of survey participants

**Survey year**		**1986**			**1989**			**1992**			**1995**			**1998**			**2001**		
		**n**	**(%)**	**Weighted (%)**	**n**	**(%)**	**Weighted (%)**	**n**	**(%)**	**Weighted (%)**	**n**	**(%)**	**Weighted (%)**	**n**	**(%)**	**Weighted (%)**	**n**	**(%)**	**Weighted (%)**
Men																			
All population (aged 25–64)		203,913			203,336			195,401			183,361			176,448			168,541		
Occupational class (EGP scheme)																			
Upper non-manual workers (I + II)		26,456	14.2	15.1	32,877	17.9	19.5	38,597	21.9	23.3	38,270	23.5	25.3	42,749	27.6	29.2	39,594	27.2	28.6
Lower non-manual workers (III)		51,095	27.4	31.0	47,502	25.8	28.4	43,706	24.8	27.1	41,904	25.7	27.9	38,696	24.9	26.4	38,373	26.3	27.9
Manual workers (V + VI + VIIa)		59,602	32.0	30.2	59,873	32.6	30.7	55,506	31.5	29.4	49,759	30.5	28.0	44,390	28.6	26.6	40,708	28.0	26.0
Farmers (IVc + VIIb)		19,529	10.5	7.4	15,857	8.6	6.2	12,040	6.8	4.9	9149	5.6	4.1	7710	5.0	3.5	6548	4.5	3.4
Self-employed (IVa + b)		29,557	15.9	16.2	27,825	15.1	15.3	26,520	15.0	15.3	23,909	14.7	14.8	21,568	13.9	14.2	20,409	14.0	14.2
Economically inactive/unknown		17,674	–	–	19,402	–	–	19,032	–	–	20,370	–	–	21,335	–	–	22,909	–	–
Women																			
All population (aged 25–64)		219,590			215,350			207,569			194,538			187,822			178,373		
Occupational class (EGP scheme)																			
Upper non-manual workers (I + II)		12,252	11.0	11.1	14,683	13.1	13.3	16,292	14.7	14.8	17,313	17.0	17.3	18,031	17.7	17.7	19,110	19.1	19.0
Lower non-manual workers (III)		48,230	43.4	48.3	49,730	44.5	48.6	52,535	47.3	51.3	50,290	49.5	53.1	52,362	51.5	54.8	53,243	53.2	55.7
Manual workers (V + VI + VIIa)		28,153	25.3	23.5	29,428	26.3	24.4	26,841	24.2	22.0	21,843	21.5	18.9	19,325	19.0	17.1	17,118	17.1	15.5
Farmers (IVc + VIIb)		15,405	13.9	10.7	11,728	10.5	8.1	8987	8.1	6.1	6435	6.3	5.0	5941	5.8	4.5	4920	4.9	4.0
Self-employed (IVa + b)		7027	6.3	6.3	6162	5.5	5.6	6328	5.7	5.7	5725	5.6	5.6	6013	5.9	6.0	5711	5.7	5.8
Economically inactive/unknown		108,523	–	–	103,619	–	–	96,586	–	–	92,932	–	–	86,150	–	–	78,271	–	–
**Survey year**		**2004**			**2007**			**2010**			**2013**			**2016**					
		**n**	**(%)**	**Weighted (%)**	**n**	**(%)**	**Weighted (%)**	**n**	**(%)**	**Weighted (%)**	**n**	**(%)**	**Weighted (%)**	**n**	**(%)**	**Weighted (%)**			
Men																			
All population (aged 25–64)		144,887			142,766			130,938			143,335			128,787					
Occupational class (EGP scheme)																			
Upper non-manual workers (I + II)		34,789	29.7	31.2	45,240	38.0	40.0	38,274	35.4	37.3	41,632	35.7	37.4	38,257	36.0	38.0			
Lower non-manual workers (III)		29,487	25.2	26.5	28,837	24.2	25.0	27,123	25.1	26.1	30,421	26.1	26.9	27,297	25.7	26.7			
Manual workers (V + VI + VIIa)		31,869	27.2	25.4	25,534	21.4	19.9	25,909	24.0	22.2	28,690	24.6	22.9	27,285	25.7	23.5			
Farmers (IVc + VIIb)		4554	3.9	2.8	4026	3.4	2.3	4159	3.8	2.7	4354	3.7	2.7	3778	3.6	2.5			
Self-employed (IVa + b)		16,352	14.0	14.2	15,423	13.0	12.8	12,608	11.7	11.7	11,558	9.9	10.2	9735	9.2	9.3			
Economically inactive/unknown		27,836	–	–	23,706	–	–	22,865	–	–	26,680	–	–	22,435	–	–			
Educational level (aged 25–64)																			
High (ISCED: 5–8)		N/A			N/A			43,165	35.3	39.3	47,687	36.3	40.3	44,372	38.9	43.2			
Middle (ISCED: 3, 4)								67,620	55.3	52.0	72,616	55.3	52.0	62,310	54.6	50.9			
Low (ISCED: 1, 2)								11,429	9.4	8.7	10,944	8.3	7.7	7384	6.5	5.9			
Educational level (aged 65–94)																			
High (ISCED: 5–8)		N/A			N/A			6745	16.1	19.6	9745	17.6	21.6	11,501	20.2	24.6			
Middle (ISCED: 3, 4)								18,179	43.3	43.3	25,575	46.2	45.6	27,819	48.8	47.4			
Low (ISCED: 1, 2)								17,026	40.6	37.0	20,068	36.2	32.8	17,703	31.0	27.9			
Women																			
All population (aged 25–64)		154,877			152,551			140,212			153,341			136,789					
Occupational class (EGP scheme)																			
Upper non-manual workers (I + II)		14,433	23.6	23.7	18,385	26.9	26.9	21,424	24.9	24.7	24,509	25.9	25.2	23,647	26.5	25.9			
Lower non-manual workers (III)		32,380	53.0	54.8	35,975	52.6	54.7	47,944	55.8	57.9	53,770	56.7	58.6	50,432	56.5	58.4			
Manual workers (V + VI + VIIa)		8975	14.7	13.0	8399	12.3	10.7	10,126	11.8	10.8	10,522	11.1	10.3	10,034	11.2	10.4			
Farmers (IVc + VIIb)		1875	3.1	2.5	1633	2.4	1.9	2633	3.1	2.3	2528	2.7	2.0	2176	2.4	1.8			
Self-employed (IVa + b)		3481	5.7	6.1	3956	5.8	5.9	3821	4.4	4.5	3479	3.7	3.9	3034	3.4	3.6			
Economically inactive/unknown		93,733	–	–	84,203	–	–	54,264	–	–	58,533	–	–	47,466	–	–			
Educational level (aged 25–64)																			
High (ISCED: 5–8)		N/A			N/A			41,362	32.3	35.5	46,178	33.5	36.6	43,571	36.7	40.1			
Middle (ISCED: 3, 4)								77,820	60.7	57.9	83,128	60.3	57.6	69,891	58.8	55.6			
Low (ISCED: 1, 2)								9020	7.0	6.6	8493	6.2	5.8	5321	4.5	4.3			
Educational level (aged 65–94)																			
High (ISCED: 5–8)		N/A			N/A			2996	5.6	6.7	4814	6.8	8.4	6114	8.7	10.6			
Middle (ISCED: 3, 4)								24,346	45.4	47.8	35,240	49.8	52.2	37,950	53.7	55.5			
Low (ISCED: 1, 2)								26,304	49.0	45.4	30,697	43.4	39.4	26,590	37.6	33.9			

*EGP scheme* Erikson-Goldthorpe-Portocarero scheme, *ISCED*: International Standard Classification of Education

Low (ISCED: 1, 2): Elementary school/junior high school graduation

Middle (ISCED: 3, 4): High school/technical professional school graduation

High (ISCED: 5–8): Two-year college/university graduation, or more

### Self-rated health in 2016

**Fig. 1:**
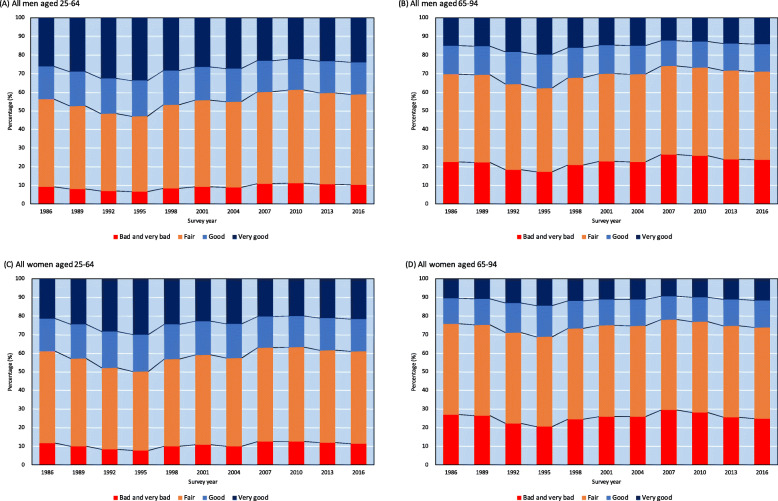
Age-adjusted percentages of self-rated health among all population between 1986 and 2016: age-adjusted were calculated with the use of proportional odds logistic regression models controlling age category (5-years) and occupational class (aged 25–64 only): estimated by Model 2

The results of all survey years are shown in Fig. 1. In 2016, the age-adjusted percentages of self-rated health (very good, good, fair, and bad/very bad) were 24.0, 17.1, 48.7, and 10.2% for working-age men, and 21.6, 17.5, 49.4, and 11.5% for working-age women, respectively. Lower self-rated health was more common in women than in men. In addition, lower self-rated health (the high percentages of “bad/very bad”) was more prominent among the elderly than among working-age men and women. In 2016, the age-adjusted percentages of self-rated health (very good, good, fair, and bad/very bad) were 14.1, 14.9, 47.4, and 23.5% for all elderly men, and 11.6, 14.6, 49.0, and 24.8%, for all elderly women, respectively.

**Fig. 2:**
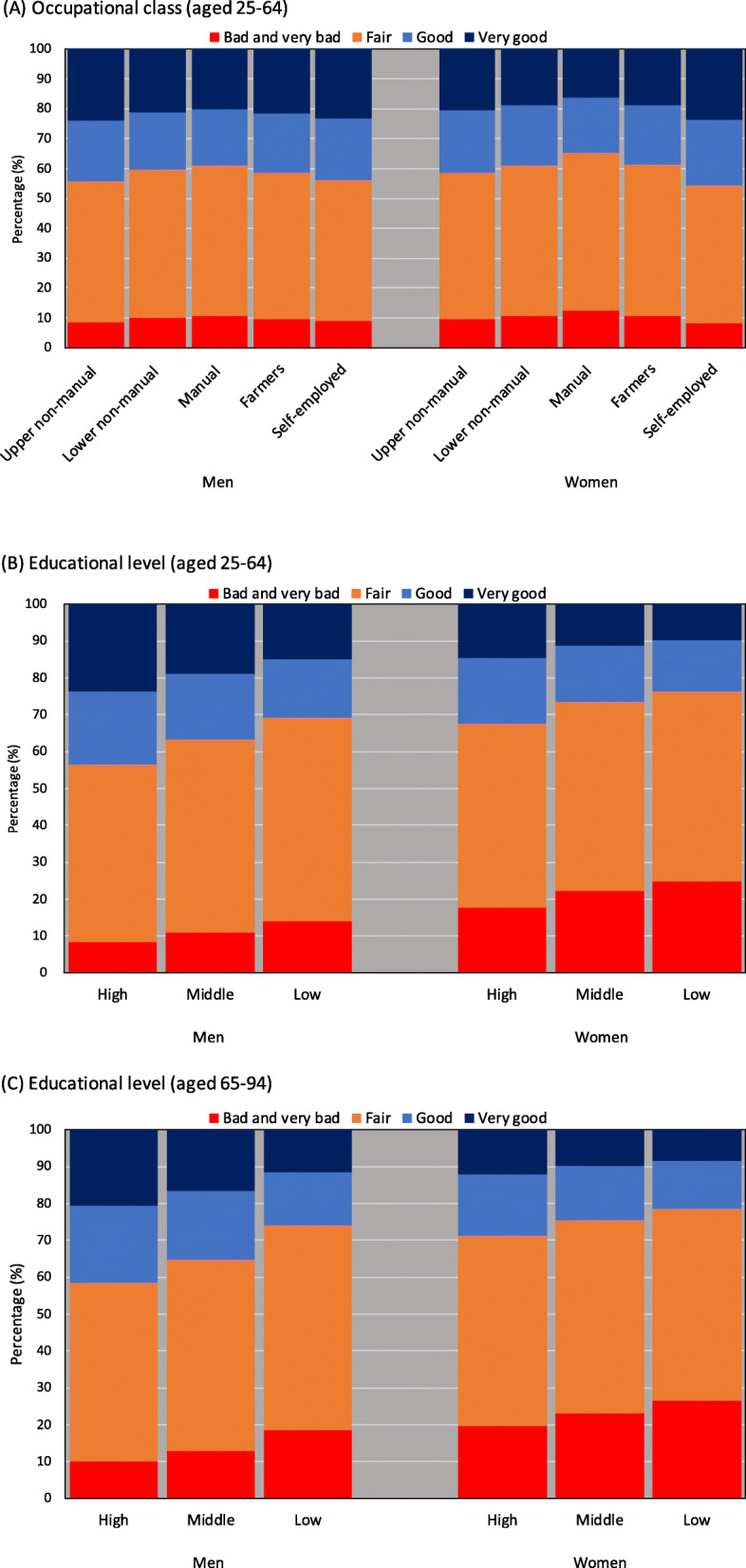
Age-adjusted percentages of self-rated health (four categories: very good, good, fair, and bad/very bad), by occupational class and educational level, in 2016. ‡ Age-adjustments were calculated using a proportional odds logistic regression model, controlling age category (5-year age interval)

Figure 2 shows the 2016 age-adjusted percentages of self-rated health by occupational class and educational level (estimated by Model 1). Although the distribution differences by occupational class are small for men, upper non-manual workers showed better distribution of self-rated health, than other occupational classes. The distribution of self-employed women was similar to upper non-manual workers (Fig. 2A). We found clear differences by educational level, and observed a better distribution of self-rated health, in order of high, middle, and low education, for both genders and age groups (Fig. 2B and C). For working-age men, the percentages of “very good” by educational level were 23.6% for “high,” 18.8% for “middle,” and 15.0% for “low,” respectively. For working-age women, percentages of “very good” by educational level were 14.5% for “high,” 11.3% for “middle,” and 9.9% for “low,” respectively. Similar patterns were observed among the elderly. For elderly men, the percentages of “very good” by educational level were 20.6% for “high,” 16.5% for “middle,” and 11.4% for “low,” respectively. For elderly women, percentages of “very good” by educational level were 12.0% for “high,” 9.9% for “middle,” and 8.4% for “low,” respectively.

### Trends in self-rated health between 1986 and 2016

Table 2 shows the overall population OR trend of less good self-rated health over the entire study period (estimated by Model 2). The OR of less good self-rated health for the overall population decreased between 1986 and 1995, and was lowest in 1995; OR for working-age men: 0.69 [95% Confidence Interval (hereafter, 95% CI): 0.66–0.71], compared with 1986. Between 1995 and 2010, the ORs of less good self-rated health increased, peaking in 2010 (OR for working-age men = 1.23 [95% CI: 1.19–1.27] and OR for working-age women = 1.10 [95% CI: 1.07–1.13], compared with 1986). After 2010, it decreased slightly; in 2016, the OR for working-age men was 1.12 (95% CI: 1.08–1.15). Therefore, two inflection points were inferred, for 1995 (lowest ORs) and 2010 (highest ORs), among working-age men and women. The temporal patterns were similar across genders and age groups, but the highest ORs were observed in 2007 for elderly men and women. Between 1995 and 2010, the ORs of less good self-rated health increased, peaking in 2007 (OR for elderly men = 1.24 [95% CI: 1.12–1.38] and OR for elderly women = 1.13 [95% CI: 1.02–1.25], compared with 1986) for elderly men and women. Please note that Fig. 1 visually illustrates the remarkable trends in Japan, especially the changes in a full spectrum of self-rated health, because these results were derived from the same analysis (Model 2).

**Table 2:** Odds ratios (ORs)^a^ of less good self-rated health among men and women, aged 25 to 64 (including occupational class) and aged 65 to 94: Pooled analysis between 1986 and 2016

	Men				Women			
	Aged 25–64		Aged 65–94		Aged 25–64		Aged 65–94	
	OR^a^	95% CI	OR^a^	95% CI	OR^a^	95% CI	OR^a^	95% CI
Survey year^b^								
1986	1.00	(Reference)	1.00	(Reference)	1.00	(Reference)	1.00	(Reference)
1989	0.86	(0.83–0.90)	0.98	(0.87–1.09)	0.85	(0.82–0.88)	0.97	(0.86–1.08)
1992	0.73	(0.70–0.76)	0.78	(0.69–0.87)	0.69	(0.66–0.71)	0.78	(0.69–0.87)
1995	0.69	(0.66–0.71)	0.71	(0.63–0.80)	0.64	(0.62–0.66)	0.69	(0.62–0.78)
1998	0.89	(0.85–0.92)	0.91	(0.81–1.01)	0.84	(0.81–0.87)	0.87	(0.78–0.97)
2001	0.98	(0.95–1.02)	1.01	(0.91–1.13)	0.93	(0.90–0.96)	0.95	(0.85–1.06)
2004	0.94	(0.91–0.98)	1.00	(0.89–1.12)	0.86	(0.83–0.89)	0.94	(0.84–1.05)
2007	1.18	(1.15–1.22)	1.24	(1.12–1.38)	1.09	(1.05–1.13)	1.13	(1.02–1.25)
2010	1.23	(1.19–1.27)	1.19	(1.09–1.31)	1.10	(1.07–1.13)	1.07	(0.98–1.18)
2013	1.15	(1.12–1.19)	1.08	(0.99–1.19)	1.03	(1.00–1.06)	0.93	(0.85–1.02)
2016	1.12	(1.08–1.15)	1.06	(0.97–1.16)	1.00	(0.97–1.03)	0.90	(0.82–0.99)
Age (5-years)								
Age (reference: 25–29 years)	1.02	(1.02–1.02)			1.02	(1.02–1.02)		
Age (reference: 65–69 years)			1.04	(1.04–1.04)			1.04	(1.04–1.04)
Occupational class (EGP scheme)								
Upper non-manual workers (I+II)	1.00	(Reference)			1.00	(Reference)		
Lower non-manual workers (III)	1.10	(1.08–1.12)			1.07	(1.06–1.09)		
Manual workers (V+VI+VIIa)	1.15	(1.13–1.18)			1.17	(1.14–1.20)		
Farmers (IVc+VIIb)	0.99	(0.95–1.02)			1.05	(1.01–1.09)		
Self-employed (IVa+b)	1.06	(1.03–1.08)			0.97	(0.94–1.00)		

*95% CI* 95% Confidence Interval, *EGP scheme* Erikson-Goldthorpe-Portocarero scheme

^a^Odds ratios (ORs) were calculated using proportional odds multiple logistic regression models, including age category (5-years), survey year (dummy variable), and occupational class (for men and women, aged 25 to 64 years) as covariates (Model 2). The odds ratio is the probability of less good self-rated health compared with the reference group or year

^b^Dummy variables of the survey year (0 = 1986, 10 = 2016) were included

### Trends in socioeconomic inequalities in self-rated health

Both favorable and unfavorable changes in self-rated health were observed among workers of all occupational classes in accordance with trends for the entire population during the study period (shown in Additional file 1: Appendix Table 4). Table 3 shows the ORs of less good self-rated health, by occupational class and educational level, for 1986, 1995, 2010, and 2016 (estimated by Model 1). The results of all surveys are shown in Additional file 1: Appendix Table 5 (for occupational class) and Additional file 1: Appendix Table 6 (for educational level). In 1986, compared to upper non-manual workers, the ORs for men ranged from 0.92 for farmers (95% CI: 0.82–1.04) to 1.15 for manual workers (95% CI: 1.06–1.23). From 1986 to 1995, we found no changes in socioeconomic inequalities in self-rated health, as evidenced by no significant interaction term between occupational class and survey year (estimated by Model 3, Additional file 1: Appendix Table 7). Between 1995 and 2010, changes were found among some socioeconomic groups as follows. The ORs of male manual workers (compared to upper non-manual workers) increased from 1.10 (95% CI: 1.03–1.18) to 1.21 (95% CI: 1.16–1.26), as evidenced by the significant interaction term. There were also changes in inequalities, by occupational class, between 2010 and 2016. In 2016, the ORs of male, lower non-manual workers (compared to upper non-manual workers) increased from 1.12 (95% CI: 1.07–1.17) in 2010 to 1.20 (95% CI: 1.15–1.26) in 2016, as evidenced by the significant interaction term. The ORs of female farmers (compared to upper non-manual workers) increased from 1.00 (95% CI: 0.90–1.11) in 2010, to 1.25 (95% CI: 1.12–1.40) in 2016, as evidenced by the significant interaction term.

**Table 3:** Odds ratios (ORs)^a^ of less good self-rated health, by occupational class and educational level, in four distinctive survey years

Survey year	1986^b^		1995^c^		2010^d^		2016^e^	
	OR^a^	95% CI	OR^a^	95% CI	OR^a^	95% CI	OR^a^	95% CI
Men								
Occupational class (EGP scheme, aged 25–64)								
Upper non-manual workers (I+II)	1.00	(Reference)	1.00	(Reference)	1.00	(Reference)	1.00	(Reference)
Lower non-manual workers (III)	1.09	(1.01–1.18)	1.06	(0.99–1.14)	1.12	(1.07–1.17)	1.20	(1.15–1.26)
Manual workers (V+VI+VIIa)	1.15	(1.06–1.23)	1.10	(1.03–1.18)	1.21	(1.16–1.26)	1.27	(1.21–1.33)
Farmers (IVc+VIIb)	0.92	(0.82–1.04)	0.95	(0.85–1.06)	1.03	(0.94–1.12)	1.18	(1.07–1.29)
Self-employed (IVa+b)	1.10	(1.02–1.19)	1.05	(0.98–1.13)	1.02	(0.97–1.08)	1.08	(1.01–1.14)
Educational level (aged 25–64)								
High (ISCED: 5–8)	N/A		N/A		1.00	(Reference)	1.00	(Reference)
Middle (ISCED: 3, 4)					1.22	(1.18–1.27)	1.34	(1.29–1.38)
Low (ISCED: 1, 2)					1.47	(1.39–1.56)	1.75	(1.63–1.88)
Educational level (aged 65–94)								
High (ISCED: 5–8)	N/A		N/A		1.00	(Reference)	1.00	(Reference)
Middle (ISCED: 3, 4)					1.34	(1.20–1.51)	1.34	(1.20–1.48)
Low (ISCED: 1, 2)					1.48	(1.31–1.66)	1.54	(1.38–1.72)
Women								
Occupational class (EGP scheme, aged 25–64)								
Upper non-manual workers (I+II)	1.00	(Reference)	1.00	(Reference)	1.00	(Reference)	1.00	(Reference)
Lower non-manual workers (III)	1.08	(1.00–1.16)	1.08	(1.02–1.15)	1.06	(1.01–1.10)	1.11	(1.07–1.16)
Manual workers (V+VI+VIIa)	1.12	(1.03–1.21)	1.14	(1.06–1.23)	1.19	(1.12–1.26)	1.34	(1.27–1.43)
Farmers (IVc+VIIb)	0.95	(0.84–1.07)	1.05	(0.94–1.19)	1.00	(0.90–1.11)	1.25	(1.12–1.40)
Self-employed (IVa+b)	1.01	(0.91–1.11)	1.03	(0.93–1.13)	0.93	(0.85–1.02)	0.86	(0.78–0.96)
Educational level (aged 25–64)								
High (ISCED: 5–8)	N/A		N/A		1.00	(Reference)	1.00	(Reference)
Middle (ISCED: 3, 4)					1.22	(1.17–1.28)	1.32	(1.26–1.37)
Low (ISCED: 1, 2)					1.74	(1.61–1.88)	2.03	(1.87–2.21)
Educational level (aged 65–94)								
High (ISCED: 5–8)	N/A		N/A		1.00	(Reference)	1.00	(Reference)
Middle (ISCED: 3, 4)					1.25	(1.09–1.43)	1.24	(1.11–1.40)
Low (ISCED: 1, 2)					1.54	(1.34–1.76)	1.49	(1.32–1.68)

*95% CI* 95% Confidence Interval, *EGP scheme* Erikson-Goldthorpe-Portocarero scheme, *ISCED* International Standard Classification of Education

Low (ISCED: 1, 2): Elementary school/junior high school graduation

Middle (ISCED: 3, 4): High school/technical professional school graduation

High (ISCED: 5–8): Two-year college/university graduation, or more

^a^ Estimates were calculated using proportional odds logistic regression models, controlling age category (5-years): Model 2. The odds ratio is the probability of less good self-rated health compared with the reference group

^b^ Year of starting the survey

^c^ Year of best self-rated health in Japan, between 1986 and 2016

^d^ Year of Japanese’ worst self-rated health for working aged men and women, between 1986 and 2016, and the year of starting the survey including educational background

^e^ The latest survey

Regarding the differences by educational level between 2010 and 2016, the ORs of working-age men with middle and low education level (compared to high education) increased from 1.22 (95% CI: 1.18–1.27) to 1.34 (95% CI: 1.29–1.38), and from 1.47 (95% CI: 1.39–1.56) to 1.75 (95% CI: 1.63–1.88), as evidenced by the significant interaction term for the interaction between educational level and survey year (estimated by Model 3 shown in Additional file 1: Appendix Table 7). The ORs of working-age, middle and low education level women, also increased from 1.22 (95% CI: 1.17–1.28) to 1.32 (95% CI: 1.26–1.37), and from 1.74 (95% CI: 1.61–1.88) to 2.03 (95% CI: 1.87–2.21) during the same period, but the interaction terms were not significant. The patterns are similar among elderly men and women, but no changes were found between 2010 and 2016. The ORs of the elderly middle and low education level men changed from 1.34 (95% CI: 1.20–1.51) to 1.34 (95% CI: 1.20–1.48), and from 1.48 (95% CI: 1.31–1.66) to 1.54 (95% CI: 1.38–1.72) during the same period. The ORs of the elderly middle and low education level women changed from 1.25 (95% CI: 1.09–1.43) to 1.24 (95% CI: 1.11–1.40), and from 1.54 (95% CI: 1.34–1.76) to 1.49 (95% CI: 1.32–1.68) during the same period.

## Discussion

### Main findings

This study incorporated the following features to overcome the limitations of previous studies: (1) we used an ordinal scale to measure self-rated health as an outcome variable [31], as opposed to dichotomized results [7–11]; (2) this study included an assessment of educational inequalities using internationally comparable classifications, which has not been previously addressed by studies in Japan, and (3) the data for the present study represents a long study period and includes a large dataset in terms of the number of survey participants included. In this study, data analysis demonstrates that Japanese self-rated health trends fluctuated during the study period. The lowest prevalence of less good self-rated health was observed in the mid-1990s, while the highest was observed around the late 2000s. We confirmed that there were socioeconomic inequalities in self-rated health among several occupational classes throughout the study period. We also found substantial and growing inequalities by educational level between 2010 and 2016. However, it should be noted that such increases were limited to particular groups, such as low- to middle-education level working-age men.

### Interpretation

The most notable finding in this study is the observation of two inflection points for self-rated health in Japan, which differs from the successive declining trend of less good self-rated health observed in other high-income countries (i.e., European countries) [32]. Previous studies have reported on the first inflection point (a reversal of the improving trends, circa 1995) [8, 9]. This study identified a second inflection point: the lowest self-ratings of health occurring in the late 2000s. One explanation is that men and women, especially those of working age, suffered the adverse health effects of Japan’s economic stagnation. In fact, some health indicators declined dramatically in the late 1990s. For example, Japan’s suicide rate surged in 1998, due to the impact of the 1997 Asian financial crisis [14]. The high suicide rate was constant until 2010, which corresponds to the period of self-rated health deterioration identified in this study (Table 2 and Fig. 1). Thus, unfavorable changes in self-rated health between 1995 and 2010 may be due to the physical and psychological state of the Japanese people, caused by the prolonged economic stagnation [7–11]. This implies self-rated health deterioration may mediate high suicide rate, especially during the economic crisis. Our findings also confirmed improvement of self-rated health across all sex and age groups after 2010 in addition to a decreasing suicide rate during the 2010s [33]. Further monitoring for self-rated health is necessary to investigate the population’s health.

Previous studies have reported that all-cause mortality among upper non-manual workers (i.e. managers and professionals) increased rapidly in the late 1990s [6, 34]. Our findings do not identify the reasons behind this trend; however, unfavorable changes in self-rated health were definitely observed among male workers of all occupational classes (Additional file 1: Appendix Table 4). Therefore, absolute self-rated health deterioration among male upper non-manual workers may be related to the rapidly increasing mortality rate during the late 1990s. This description implies that self-rated health deterioration across the Japanese population may have reflected the deterioration of health and may partially explain further deviations from the typical pattern of mortality inequalities, as observed in European countries [6]. Although we found that upper non-manual workers had better self-rated health throughout the study period, the discrepancy between favorable self-rated health but high mortality among male upper non-manual workers, represents a unique pattern among Japanese working-age men.

We should carefully interpret the discrepancy between trends in Japanese individuals’ self-rated health and mortality rate in this period. Assuming that less good self-rated health is associated with high mortality risk, mortality will continue to increase, as per the observed high prevalence of less good self-rated health in Japan between 1995 and 2010. However, the Japanese age-adjusted mortality rate declined during this period [2]. One possible reason is that there are many drivers of and competing factors in mortality, indicative of the weak relationship between self-rated health and mortality among the Japanese population.

Another important finding of this study is that regarding inequalities in self-rated health by occupational class, which remained stable and relatively small throughout the study period. This finding is consistent with previous studies on the trends in Japan between 1986 and 2007 [8, 9]. In addition to these trends, we found that socioeconomic inequalities have increased since 2007 between certain socioeconomic groups, especially those of working age. Therefore, we recommend a careful assessment to monitor self-rated health inequalities in line with occupational health.

We have identified distinct differences in self-rated health across educational levels in Japan, which is a novel finding. The magnitude of inequalities among working-age men and women was more pronounced by educational level than by occupational class, which is consistent with the trends observed in European countries and South Korea [31, 35]. However, educational inequalities in self-rated health should be interpreted with caution, because in Japan, the proportion of working-age men and women with a low education level is very small (5.9% for men, and 4.3% for women, in 2016). Nevertheless, we consider educational inequalities in self-rated health among the elderly as a particularly serious issue because the proportions of elderly men and women with a low education level were substantial (27.9 and 33.9%, respectively, in 2016), and the prevalence of less good self-rated health (bad or very bad at 24.8% for women aged 65 to 94) is larger than that for the working-age group (11.5% for women). As the Japanese society ages, health promotion is becoming increasingly important for the latter part of individuals’ lives. Although we need a careful assessment of the features of each cohort on self-rated health and health inequalities [10, 11], our findings shed light on socioeconomic inequalities in the health of the elderly in a super-aged society.

Our analysis may facilitate between-country comparative research on self-rated health, because the symmetric response scale (five-point scale) affords easy comparisons with other high-income countries, especially in European countries [13, 32]. Such international comparative study can focus on the magnitude of socioeconomic inequalities in Japan and the difference in the meaning of the category “fair” compared with other high-income countries. In addition, the unique Japanese trends in self-rated health can be contrasted to neighbouring Asian countries (e.g. South Korea) that have both cultural similarities and differences [35, 36].

### Strengths and limitations

This study builds on previous research on health inequalities and is based on a long-term, nationwide survey and a comprehensive assessment of socioeconomic inequalities in Japan. Whereas the previous studies used dichotomized self-rated health as an outcome [7–11]. 

We adopted four categories: very good, good, fair, and bad/very bad. This study also included the trends in health inequalities at the educational level, which has not been addressed in previous studies [8, 9].

However, self-rated health for the Japanese population should be interpreted with caution because the Japanese are generally more likely to choose the mid-point (fair) when they respond to a Likert-type scale survey [37]. For example, for the year 2016, we estimated that approximately 50% of people in Japan perceived their health as fair (based on age-adjusted percentages calculated by model 2). Assuming that we define the prevalence of “less than good health” as an outcome for assessing self-rated health in 2016, the prevalence was 58.9% for working-age men (Fig. 1). This is relatively higher than in Western and Southern European countries (15 to 45%, for men aged 30 to 79) [32]. This may be because the Japanese positively interpret the word “futsu” (fair, in Japanese), whereas westerners consider “fair” literally, as a neutral position [37]. This means that the results from Japan may lean more toward less good self-rated health than those from other high-income countries [38]. Therefore, Japanese socioeconomic inequalities in self-rated health may be systematically underestimated, supposing that the cultural tendencies to choose the mid-point of Likert scales have not changed during the study period. Further research is required to show whether the Japanese perception of “fair” health is homogenous in terms of objective health status.

Another limitation is the parallel assumptions for the ordinal logistic regression model. Our analysis suggests that these assumptions were violated. A sensitive analysis (binary logistic regression models using “less than good health” and “bad/very bad” as outcomes, respectively) confirmed similar results to those presented in this study. For working-age men, for example, the prevalence of “less than good health” by educational level were 56.5% for “high,” 63.3% for “middle,” and 69.3% for “low,” respectively (shown in 2B) whereas the prevalence calculated by binary logistic regression models (sensitive analysis) were 55.8% for “high,” 63.9% for “middle,” and 68.8% for “low,” respectively. For elderly women, the prevalence of “less than good health” by educational level were 71.2% for “high,” 75.3% for “middle,” and 78.5% for “low,” respectively (shown in 2C) whereas the prevalence calculated by binary logistic regression models (sensitive analysis) were 69.6% for “high,” 75.1% for “middle,” and 79.0% for “low,” respectively. These data confirm that the application of ordinal logistic regression models did not distort the overall trends of the analysis.

## Conclusion

Japan had relatively poor self-rated health because of the high prevalence (about 50%) of “fair” (i.e., the mid-point) in self-rated health, which was a unique feature of survey response patterns and a key feature for better understanding trends in self-rated health in the Japanese population. We found important variations over time and between socioeconomic groups, and distinct differences in self-rated health by educational level, which have increased since 2010 whereas those by occupational class were stable and relatively small throughout the study period. Therefore, our study results suggest that socioeconomic inequalities in health in Japan are more similar to those in other high-income countries than often assumed. In-depth studies of the role of socioeconomic conditions may shed light on the reasons for the high prevalence of poor self-rated health in Japan. In addition, a better understanding of the high prevalence of “fair (futsu)” is a key element for elucidating the overall picture of self-rated health in Japan.

## Supplementary Information

**Additional file 1.**

## Data Availability

No additional data are available in this study. However, data are available with the permission of the Ministry of Health, Labor and Welfare.
