# Supplementary material for: Trends and socioeconomic inequalities in self-rated health in Japan, 1986–2016
Source: BMC Public Health. 2021 Oct 8;21:1811. doi: 10.1186/s12889-021-11708-6 (PMC8501722; doi:10.1186/s12889-021-11708-6)
Supplement: Supplementary file 1 — Additional file 1. [file 12889_2021_11708_MOESM1_ESM.docx]

**Supplementary file**

**Trends and socioeconomic inequalities in self-rated health in Japan, 1986**–**2016**

Hirokazu Tanaka;^1,2, 3^ Johan P. Mackenbach;^1^ Yasuki Kobayashi^3^*

^1^ Department of Public Health, Erasmus University Medical Center, 3000 CA Rotterdam, The Netherlands.

^2^ Department of Public Health and Occupational Medicine, Graduate School of Medicine, Mie University, 514-8507 Mie, Japan.

^3^ Department of Public Health, Graduate School of Medicine, The University of Tokyo, 113-0033 Tokyo, Japan.

***Corresponding author:** Yasuki Kobayashi

Department of Public Health, Graduate School of Medicine, The University of Tokyo, Tokyo, Japan

7-3-1 Hongo, Bunkyo-ku, Tokyo 113-0033, Japan

E-mail: [yasukik@m.u-tokyo.ac.jp](mailto:yasukik@m.u-tokyo.ac.jp)

**Contents:**

**Appendix Table 1.** Definitions of occupational class (page 3)

**Appendix Table 2.** Definitions of educational level (page 4)

**Appendix Table 3.** Distributions of educational level by occupational class (page 5)

**Appendix Table 4.** Trends in odds ratios (ORs) of less good self-rated health compared with 1986, by occupational class (page 6-7)

**Appendix Table 5.** Trends in odds ratios (ORs) of less good self-rated health by occupational class (EGP scheme) among men and women aged 25-64 (page 8-9)

**Appendix Table 6.** Odds ratios (ORs) of less good self-rated health by educational level

(page 10)

**Appendix Table 7.** Interaction terms of proportional odds multiple logistic regression models between occupational class (educational class) and survey year by three period (1986-1995, 1995-2010, and 2010-2016) (page 11-12)

| **Appendix Table 1.** Definitions of occupational class | | | |
| --- | --- | --- | --- |
| Occupational class | Occupation defined by Japanese Standard Occupational Classification (JSOC) | Employment status | Correspondence to the Erikson-Goldthorpe-Portocarero (EGP) scheme |
| Upper non-manual workers | (A) Administrative and managerial workers | - | I: Higher-grade professionals, administrators and officials; managers in large industrial establishments; large proprietors |
|  | (B) Professional and engineering workers | - |  |
|  |  | - | II: Lower-grade professionals, administrators and officials; higher-grade technicians; managers in small business and industrial establishments; supervisors of non-manual employees |
| Lower non-manual workers | (C) Clerical workers | - | III: Routine non-manual employees in administration and commerce; sales personnel; other rank-and-file service workers |
|  | (D) Sales workers | - |  |
|  | (E) Service workers |  |  |
| Manual workers | (H) Manufacturing process workers | - | V/VI: Lower-grade technicians; supervisors of manual workers; skilled manual workers |
|  | (I) Transport and machine operating workers | - |  |
|  | (J) Construction and mining workers | - |  |
|  | (K) Carrying, cleaning, packaging, and related workers | - | VIIa: Semi- and unskilled manual workers (not in agriculture) |
| Farmers | (G) Agriculture forestry and fishery workers | - | IVc: Farmers and smallholders; self-employed fishermen  VIIb: Agricultural workers |
| Self-employed | - | Self-employed with employees, self-employed no employees | IVa: Small proprietors; artisans, etc., with employees  IVb: Small proprietors, artisans, etc., without employees |

| **Appendix Table 2.** Definitions of educational level | | | |
| --- | --- | --- | --- |
| Educational level | Educational background  defined by Japanese national surveys | Correspondence to the International Standard  Classification of Education (ISCED), 1997 | Correspondence to the International Standard  Classification of Education (ISCED), 2011 |
| Low | Elementary school/Junior high school graduates | ISCED 1: Primary education | ISCED 1: Primary education |
|  |  | ISCED 2: Lower secondary education | ISCED 2: Lower secondary education |
| Middle | High school graduates | ISCED 3: Upper secondary education | ISCED 3: Upper secondary education |
|  | Technical professional school graduates | ISCED 4: Post-secondary non-tertiary education | ISCED 4: Post-secondary non-tertiary education |
| High | 2-year college graduates | ISCED 5: First stage of tertiary education | ISCED 5: Short-cycle tertiary education |
|  | University graduates | ISCED 6: Second stage of tertiary education | ISCED 6: Bachelor’s or equivalent level |
|  | Graduate school |  | ISCED 7: Master’s or equivalent level |
|  |  |  | ISCED 8: Doctoral or equivalent level |

| **Appendix Table 3.** Distributions of educational level by occupational class (EGP scheme, aged 25-64) | | | | | | | | | |
| --- | --- | --- | --- | --- | --- | --- | --- | --- | --- |
|  | 2010 | | | 2013 | | | 2016 | | |
|  | Low (ISCED:  1, 2) | Middle (ISCED:  3, 4) | High (ISCED:  5-8) | Low (ISCED:  1, 2) | Middle (ISCED:  3, 4) | High (ISCED:  5-8) | Low (ISCED:  1, 2) | Middle (ISCED:  3, 4) | High (ISCED:  5-8) |
| Men |  |  |  |  |  |  |  |  |  |
| Upper non-manual workers (I+II) | 2.8 | 40.3 | 57.0 | 2.2 | 39.8 | 57.9 | 1.7 | 38.3 | 60.0 |
| Lower non-manual workers (III) | 3.8 | 48.7 | 47.5 | 3.3 | 47.7 | 49.0 | 2.4 | 45.6 | 52.0 |
| Manual workers (V+VI+VIIa) | 16.2 | 69.9 | 13.9 | 13.8 | 70.9 | 15.2 | 11.3 | 71.2 | 17.5 |
| Farmers (IVc+VIIb) | 15.4 | 65.3 | 19.2 | 14.2 | 67.3 | 18.5 | 10.7 | 65.3 | 23.9 |
| Self-employed (IVa+b) | 13.4 | 56.0 | 30.6 | 12.9 | 56.7 | 30.4 | 11.4 | 56.9 | 31.7 |
| Women |  |  |  |  |  |  |  |  |  |
| Upper non-manual workers (I+II) | 1.5 | 46.1 | 52.4 | 1.5 | 46.0 | 52.5 | 1.1 | 43.7 | 55.3 |
| Lower non-manual workers (III) | 4.4 | 60.6 | 35.1 | 3.8 | 59.7 | 36.5 | 2.9 | 57.9 | 39.2 |
| Manual workers (V+VI+VIIa) | 15.0 | 73.0 | 12.0 | 12.4 | 73.0 | 14.5 | 10.1 | 74.2 | 15.7 |
| Farmers (IVc+VIIb) | 16.2 | 69.2 | 14.6 | 12.4 | 71.6 | 16.0 | 7.6 | 71.7 | 20.8 |
| Self-employed (IVa+b) | 7.0 | 55.7 | 37.3 | 5.6 | 55.1 | 39.2 | 4.2 | 51.5 | 44.4 |
| EGP scheme: Erikson-Goldthorpe-Portocarero scheme, ISCED: International Standard Classification of Education | | | | | | | | | |
| Low (ISCED: 1, 2): Elementary school/junior high school graduation | | | | | | | | | |
| Middle (ISCED: 3, 4): High school/technical professional school graduation | | | | | | | | | |
| High (ISCED: 5-8): Two-year college/university graduation, or more | | | | | | | | | |

| **Appendix Table 4.** Trends in odds ratios (ORs)^a^ of less good self-rated health compared with 1986, by occupational class (aged 25–64) | | | | | | | | | | | | | | | | | | | | |
| --- | --- | --- | --- | --- | --- | --- | --- | --- | --- | --- | --- | --- | --- | --- | --- | --- | --- | --- | --- | --- |
|  | Upper non-manual workers (I+II) | | | | Lower non-manual workers (III) | | | | Manual workers (V+VI+VIIa) | | | | Farmers  (IVc+VIIb) | | | | Self-employed (IVa+b) | | | |
|  | OR | 95% CI | | | OR | 95% CI | | | OR | 95% CI | | | OR | 95% CI | | | OR | 95% CI | | |
| Men |  |  |  |  |  |  |  |  |  |  |  |  |  |  |  |  |  |  |  |  |
| 1986 | Reference | | | | Reference | | | | Reference | | | | Reference | | | | Reference | | | |
| 1989 | 0.90 | 0.83 | - | 0.98 | 0.85 | 0.79 | - | 0.91 | 0.83 | 0.78 | - | 0.89 | 0.98 | 0.85 | - | 1.13 | 0.86 | 0.80 | - | 0.93 |
| 1992 | 0.74 | 0.68 | - | 0.80 | 0.71 | 0.66 | - | 0.77 | 0.72 | 0.67 | - | 0.77 | 0.82 | 0.71 | - | 0.94 | 0.73 | 0.68 | - | 0.79 |
| 1995 | 0.70 | 0.65 | - | 0.76 | 0.67 | 0.63 | - | 0.72 | 0.67 | 0.63 | - | 0.72 | 0.76 | 0.66 | - | 0.88 | 0.69 | 0.64 | - | 0.75 |
| 1998 | 0.89 | 0.82 | - | 0.96 | 0.87 | 0.81 | - | 0.93 | 0.88 | 0.82 | - | 0.94 | 1.00 | 0.87 | - | 1.14 | 0.87 | 0.81 | - | 0.94 |
| 2001 | 1.01 | 0.93 | - | 1.08 | 0.95 | 0.89 | - | 1.01 | 0.96 | 0.90 | - | 1.03 | 1.19 | 1.04 | - | 1.37 | 0.97 | 0.90 | - | 1.04 |
| 2004 | 0.97 | 0.90 | - | 1.04 | 0.96 | 0.90 | - | 1.02 | 0.91 | 0.86 | - | 0.97 | 1.00 | 0.86 | - | 1.15 | 0.89 | 0.82 | - | 0.96 |
| 2007 | 1.18 | 1.10 | - | 1.27 | 1.17 | 1.10 | - | 1.24 | 1.23 | 1.16 | - | 1.30 | 1.41 | 1.23 | - | 1.61 | 1.10 | 1.02 | - | 1.19 |
| 2010 | 1.24 | 1.15 | - | 1.32 | 1.23 | 1.16 | - | 1.31 | 1.25 | 1.18 | - | 1.32 | 1.39 | 1.22 | - | 1.57 | 1.15 | 1.07 | - | 1.23 |
| 2013 | 1.15 | 1.07 | - | 1.23 | 1.14 | 1.07 | - | 1.21 | 1.20 | 1.14 | - | 1.27 | 1.29 | 1.14 | - | 1.47 | 1.08 | 1.00 | - | 1.16 |
| 2016 | 1.08 | 1.01 | - | 1.16 | 1.15 | 1.08 | - | 1.22 | 1.14 | 1.08 | - | 1.20 | 1.37 | 1.20 | - | 1.55 | 1.04 | 0.97 | - | 1.12 |
| Women |  |  |  |  |  |  |  |  |  |  |  |  |  |  |  |  |  |  |  |  |
| 1986 | Reference | | | | Reference | | | | Reference | | | | Reference | | | | Reference | | | |
| 1989 | 0.83 | 0.76 | - | 0.91 | 0.81 | 0.77 | - | 0.86 | 0.87 | 0.81 | - | 0.93 | 0.94 | 0.81 | - | 1.08 | 0.92 | 0.83 | - | 1.03 |
| 1992 | 0.70 | 0.64 | - | 0.76 | 0.67 | 0.64 | - | 0.71 | 0.69 | 0.64 | - | 0.74 | 0.77 | 0.66 | - | 0.89 | 0.66 | 0.59 | - | 0.74 |
| 1995 | 0.63 | 0.57 | - | 0.68 | 0.62 | 0.59 | - | 0.66 | 0.64 | 0.59 | - | 0.69 | 0.72 | 0.62 | - | 0.83 | 0.67 | 0.60 | - | 0.74 |
| 1998 | 0.83 | 0.77 | - | 0.90 | 0.82 | 0.78 | - | 0.87 | 0.83 | 0.77 | - | 0.90 | 0.91 | 0.79 | - | 1.05 | 0.86 | 0.78 | - | 0.96 |
| 2001 | 0.92 | 0.84 | - | 0.99 | 0.91 | 0.86 | - | 0.95 | 0.93 | 0.87 | - | 1.00 | 1.07 | 0.94 | - | 1.22 | 0.90 | 0.81 | - | 0.99 |
| 2004 | 0.88 | 0.81 | - | 0.96 | 0.84 | 0.80 | - | 0.88 | 0.88 | 0.81 | - | 0.95 | 0.88 | 0.73 | - | 1.06 | 0.78 | 0.69 | - | 0.87 |
| 2007 | 1.10 | 1.01 | - | 1.18 | 1.05 | 1.00 | - | 1.11 | 1.14 | 1.06 | - | 1.23 | 1.29 | 1.11 | - | 1.51 | 0.99 | 0.89 | - | 1.10 |
| 2010 | 1.10 | 1.03 | - | 1.19 | 1.07 | 1.02 | - | 1.12 | 1.15 | 1.07 | - | 1.24 | 1.14 | 0.99 | - | 1.30 | 1.02 | 0.91 | - | 1.13 |
| 2013 | 1.00 | 0.93 | - | 1.07 | 1.01 | 0.97 | - | 1.05 | 1.14 | 1.06 | - | 1.22 | 1.11 | 0.97 | - | 1.27 | 0.89 | 0.80 | - | 1.00 |
| 2016 | 0.97 | 0.90 | - | 1.04 | 0.98 | 0.93 | - | 1.02 | 1.11 | 1.03 | - | 1.18 | 1.19 | 1.04 | - | 1.37 | 0.83 | 0.74 | - | 0.93 |
| ^a^ Estimates by survey year were calculated with the use of proportional odds logistic regression models controlling age category (5-years): estimated by model 2 | | | | | | | | | | | | | | | | | | | | |
|  |  |  |  |  |  |  |  |  |  |  |  |  |  |  |  |  |  |  |  |  |
| EGP scheme: Erikson-Goldthorpe-Portocarero scheme | | | | | | | | | | | | | | | | | | | | |
| 95% CI: 95% confidence interval | | | | | | | | | | | | | | | | | | | | |

| **Appendix Table 5.** Trends in odds ratios (ORs)^a^ of less good self-rated health by occupational class (EGP scheme) among men and women aged 25-64 | | | | | | | | | | | | | | | | | | | | | |
| --- | --- | --- | --- | --- | --- | --- | --- | --- | --- | --- | --- | --- | --- | --- | --- | --- | --- | --- | --- | --- | --- |
|  |  | Upper non-manual workers (I+II)* | | | | Lower non-manual workers (III)* | | | | Manual workers (V+VI+VIIa)* | | | | Farmers  (IVc+VIIb)* | | | | Self-employed  (IVa+b)* | | | |
|  | Year | OR | 95% CI | | | OR | 95% CI | | | OR | 95% CI | | | OR | 95% CI | | | OR | 95% CI | | |
| Men | |  |  |  |  |  |  |  |  |  |  |  |  |  |  |  |  |  |  |  |  |
|  | 1986 | Reference | | | | 1.09 | (1.01 | - | 1.18) | 1.15 | (1.06 | - | 1.23) | 0.92 | (0.82 | - | 1.04) | 1.10 | (1.02 | - | 1.19) |
|  | 1989 | Reference | | | | 1.05 | (0.98 | - | 1.12) | 1.07 | (1.00 | - | 1.15) | 0.95 | (0.85 | - | 1.06) | 1.02 | (0.95 | - | 1.10) |
|  | 1992 | Reference | | | | 1.07 | (1.00 | - | 1.15) | 1.12 | (1.05 | - | 1.20) | 0.97 | (0.87 | - | 1.08) | 1.07 | (0.99 | - | 1.15) |
|  | 1995 | Reference | | | | 1.06 | (0.99 | - | 1.14) | 1.10 | (1.03 | - | 1.18) | 0.95 | (0.85 | - | 1.06) | 1.05 | (0.98 | - | 1.13) |
|  | 1998 | Reference | | | | 1.08 | (1.02 | - | 1.15) | 1.15 | (1.08 | - | 1.22) | 1.00 | (0.91 | - | 1.11) | 1.07 | (0.99 | - | 1.15) |
|  | 2001 | Reference | | | | 1.05 | (1.00 | - | 1.11) | 1.12 | (1.06 | - | 1.19) | 1.06 | (0.96 | - | 1.17) | 1.05 | (0.98 | - | 1.12) |
|  | 2004 | Reference | | | | 1.10 | (1.05 | - | 1.16) | 1.10 | (1.04 | - | 1.16) | 0.92 | (0.82 | - | 1.03) | 1.00 | (0.93 | - | 1.07) |
|  | 2007 | Reference | | | | 1.11 | (1.06 | - | 1.17) | 1.23 | (1.17 | - | 1.30) | 1.07 | (0.97 | - | 1.19) | 1.01 | (0.94 | - | 1.08) |
|  | 2010 | Reference | | | | 1.12 | (1.07 | - | 1.17) | 1.21 | (1.16 | - | 1.26) | 1.03 | (0.94 | - | 1.12) | 1.02 | (0.97 | - | 1.08) |
|  | 2013 | Reference | | | | 1.12 | (1.07 | - | 1.17) | 1.26 | (1.21 | - | 1.31) | 1.04 | (0.95 | - | 1.14) | 1.05 | (0.99 | - | 1.11) |
|  | 2016 | Reference | | | | 1.20 | (1.15 | - | 1.26) | 1.27 | (1.21 | - | 1.33) | 1.18 | (1.07 | - | 1.29) | 1.08 | (1.01 | - | 1.14) |
| Women | |  |  |  |  |  |  |  |  |  |  |  |  |  |  |  |  |  |  |  |  |
|  | 1986 | Reference | | | | 1.08 | (1.00 | - | 1.16) | 1.12 | (1.03 | - | 1.21) | 0.95 | (0.84 | - | 1.07) | 1.01 | (0.91 | - | 1.11) |
|  | 1989 | Reference | | | | 1.05 | (0.99 | - | 1.13) | 1.15 | (1.07 | - | 1.24) | 1.01 | (0.90 | - | 1.13) | 1.07 | (0.97 | - | 1.18) |
|  | 1992 | Reference | | | | 1.04 | (0.98 | - | 1.11) | 1.09 | (1.01 | - | 1.17) | 1.00 | (0.89 | - | 1.13) | 0.91 | (0.83 | - | 1.01) |
|  | 1995 | Reference | | | | 1.08 | (1.02 | - | 1.15) | 1.14 | (1.06 | - | 1.23) | 1.05 | (0.94 | - | 1.19) | 1.03 | (0.93 | - | 1.13) |
|  | 1998 | Reference | | | | 1.08 | (1.01 | - | 1.15) | 1.13 | (1.05 | - | 1.21) | 1.04 | (0.93 | - | 1.16) | 1.04 | (0.94 | - | 1.14) |
|  | 2001 | Reference | | | | 1.08 | (1.02 | - | 1.15) | 1.16 | (1.08 | - | 1.24) | 1.12 | (1.01 | - | 1.24) | 0.99 | (0.90 | - | 1.08) |
|  | 2004 | Reference | | | | 1.03 | (0.97 | - | 1.09) | 1.13 | (1.05 | - | 1.22) | 0.98 | (0.83 | - | 1.16) | 0.90 | (0.81 | - | 1.00) |
|  | 2007 | Reference | | | | 1.05 | (0.99 | - | 1.10) | 1.17 | (1.09 | - | 1.25) | 1.12 | (0.98 | - | 1.29) | 0.89 | (0.81 | - | 0.98) |
|  | 2010 | Reference | | | | 1.05 | (0.99 | - | 1.10) | 1.19 | (1.12 | - | 1.26) | 1.00 | (0.90 | - | 1.11) | 0.93 | (0.85 | - | 1.02) |
|  | 2013 | Reference | | | | 1.12 | (1.08 | - | 1.16) | 1.34 | (1.26 | - | 1.42) | 1.13 | (1.02 | - | 1.26) | 0.92 | (0.83 | - | 1.01) |
|  | 2016 | Reference | | | | 1.11 | (1.07 | - | 1.16) | 1.34 | (1.27 | - | 1.43) | 1.25 | (1.12 | - | 1.40) | 0.86 | (0.78 | - | 0.96) |
| ^a^ Estimates were calculated with the use of proportional odds logistic regression models controlling age category (5-years) by survey year: estimated by model 1. | | | | | | | | | | | | | | | | | | | | | |
|  |  |  |  |  |  |  |  |  |  |  |  |  |  |  |  |  |  |  |  |  |  |
| EGP scheme: Erikson-Goldthorpe-Portocarero scheme | | | | | | | | | | | | | | | | | | | | | |
| 95% CI: 95% Confidence Interval | | | | | | | | | | | | | | | | | | | | | |

| **Appendix Table 6.** Odds ratios (ORs)^a^ of less good self-rated health by educational level | | | | | | | | | | | | | | | | | |
| --- | --- | --- | --- | --- | --- | --- | --- | --- | --- | --- | --- | --- | --- | --- | --- | --- | --- |
|  |  |  | High (ISCED: 5-8) | | | | | Middle (ISCED: 3, 4) | | | | | Low (ISCED: 1, 2) | | | | |
|  |  | Year | (%)^b^ | OR | 95% CI | | | (%)^b^ | OR | 95% CI | | | (%)^b^ | OR | 95% CI | | |
| Aged 25-64 | | | |  |  |  |  |  |  |  |  |  |  |  |  |  |  |
|  | Men | |  |  |  |  |  |  |  |  |  |  |  |  |  |  |  |
|  |  | 2010 | (39.3) | Reference | | | | (52.0) | 1.22 | (1.18 | - | 1.27) | (8.7) | 1.47 | (1.39 | - | 1.56) |
|  |  | 2013 | (40.3) | Reference | | | | (52.0) | 1.25 | (1.21 | - | 1.30) | (7.7) | 1.59 | (1.49 | - | 1.70) |
|  |  | 2016 | (43.2) | Reference | | | | (50.9) | 1.34 | (1.29 | - | 1.38) | (5.9) | 1.75 | (1.63 | - | 1.88) |
|  | Women | |  |  |  |  |  |  |  |  |  |  |  |  |  |  |  |
|  |  | 2010 | (35.5) | Reference | | | | (57.9) | 1.22 | (1.17 | - | 1.28) | (6.6) | 1.74 | (1.61 | - | 1.88) |
|  |  | 2013 | (36.6) | Reference | | | | (57.6) | 1.28 | (1.22 | - | 1.33) | (5.8) | 1.86 | (1.73 | - | 2.01) |
|  |  | 2016 | (40.1) | Reference | | | | (55.6) | 1.32 | (1.26 | - | 1.37) | (4.3) | 2.03 | (1.87 | - | 2.21) |
| Aged 65-94 | | | |  |  |  |  |  |  |  |  |  |  |  |  |  |  |
|  | Men | |  |  |  |  |  |  |  |  |  |  |  |  |  |  |  |
|  |  | 2010 | (19.6) | Reference | | | | (43.3) | 1.34 | (1.20 | - | 1.51) | (37.0) | 1.48 | (1.31 | - | 1.66) |
|  |  | 2013 | (21.6) | Reference | | | | (45.6) | 1.26 | (1.12 | - | 1.40) | (32.8) | 1.50 | (1.34 | - | 1.68) |
|  |  | 2016 | (24.6) | Reference | | | | (47.4) | 1.34 | (1.20 | - | 1.48) | (27.9) | 1.54 | (1.38 | - | 1.72) |
|  | Women | |  |  |  |  |  |  |  |  |  |  |  |  |  |  |  |
|  |  | 2010 | (6.7) | Reference | | | | (47.8) | 1.25 | (1.09 | - | 1.43) | (45.4) | 1.54 | (1.34 | - | 1.76) |
|  |  | 2013 | (8.4) | Reference | | | | (52.2) | 1.24 | (1.09 | - | 1.41) | (39.4) | 1.45 | (1.28 | - | 1.65) |
|  |  | 2016 | (10.6) | Reference | | | | (55.5) | 1.24 | (1.11 | - | 1.40) | (33.9) | 1.49 | (1.32 | - | 1.68) |
| ^a^ Estimates were calculated with the use of proportional odds logistic regression models controlling age category (5-years) by survey year: estimated by model 1. | | | | | | | | | | | | | | | | | |
| ^b^ Population percentage | | | | | | | | | | | | | | | | | |
| ISCED: International Standard Classification of Education | | | | | | | | | | | | | | | | | |
| Low (ISCED: 1, 2): Elementary school/junior high school graduation | | | | | | | | | | | | | | | | | |
| Middle (ISCED: 3, 4): High school/technical professional school graduation | | | | | | | | | | | | | | | | | |
| High (ISCED: 5-8): Two-year college/university graduation, or more | | | | | | | | | | | | | | | | | |
| 95% CI: 95% Confidence Interval | | | | | | | | | | | | | | | | | |

| **Appendix Table 7.** Interaction terms of proportional odds multiple logistic regression models^a^ between occupational class (EGP scheme) and educational level and survey year by three period (1986-1995, 1995-2010, and 2010-2016) | | | | |
| --- | --- | --- | --- | --- |
|  |  |  | Men | Women |
|  |  |  | Coefficient^a^ | Coefficient^a^ |
| Interaction term (occupational class*survey year)^b^ | | |  |  |
|  | Between 1986-1995 (aged 25-64) | |  |  |
|  |  | Upper non-manual workers (I+II)*survey year | Reference | Reference |
|  |  | Lower non-manual workers (III)*survey year | -0.011 | 0.000 |
|  |  | Manual workers (V+VI+VIIa)*survey year | -0.011 | 0.000 |
|  |  | Farmers (IVc+VIIb)*survey year | 0.022 | 0.036 |
|  |  | Self-employed (IVa+b)*survey year | -0.008 | -0.007 |
|  | Between 1995-2010 (aged 25-64) | |  |  |
|  |  | Upper non-manual workers (I+II)*survey year | Reference | Reference |
|  |  | Lower non-manual workers (III)*survey year | 0.011 | -0.008 |
|  |  | Manual workers (V+VI+VIIa)*survey year | 0.017* | 0.009 |
|  |  | Farmers (IVc+VIIb)*survey year | 0.019 | -0.003 |
|  |  | Self-employed (IVa+b)*survey year | -0.007 | -0.030* |
|  | Between 2010-2016 (aged 25-64) | |  |  |
|  |  | Upper non-manual workers (I+II)*survey year | Reference | Reference |
|  |  | Lower non-manual workers (III)*survey year | 0.037* | 0.021 |
|  |  | Manual workers (V+VI+VIIa)*survey year | 0.021 | 0.047* |
|  |  | Farmers (IVc+VIIb)*survey year | 0.060 | 0.091* |
|  |  | Self-employed (IVa+b)*survey year | 0.018 | -0.049 |
| Interaction term (Educational level*survey year)^c^ | | |  |  |
|  | Between 2010-2016 (aged 25-64) | |  |  |
|  |  | High (ISCED: 5-8)*survey year | Reference | Reference |
|  |  | Middle (ISCED: 3, 4)*survey year | 0.039* | 0.028 |
|  |  | Low (ISCED: 1, 2)*survey year | 0.075* | 0.050 |
|  | Between 2010-2016 (aged 65-94) | |  |  |
|  |  | High (ISCED: 5-8)*survey year | Reference | Reference |
|  |  | Middle (ISCED: 3, 4)*survey year | -0.007 | -0.013 |
|  |  | Low (ISCED: 1, 2)*survey year | -0.005 | -0.038 |
| * p < 0.05 | | | | |
| ^a^ Coefficients were calculated with the use of proportional odds multiple logistic regression models adjusted age category (5-years) and survey year including an interaction term between occupational class (educational class) and survey year: estimated by model 3. | | | | |
| ^b^ Dummy variables of survey year (0: the year of 1986 to 10: the year of 2016) were included as a continuous variable. | | | | |
| ^c^ Dummy variables of survey year (0: the year of 2010 to 2: the year of 2016) were included as a continuous variable. | | | | |
| EGP scheme: Erikson-Goldthorpe-Portocarero scheme, ISCED: International Standard Classification of Education | | | | |
| Low (ISCED: 1, 2): Elementary school/junior high school graduation | | | | |
| Middle (ISCED: 3, 4): High school/technical professional school graduation | | | | |
| High (ISCED: 5-8): Two-year college/university graduation, or more | | | | |
